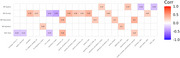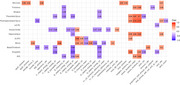# Mapping Associations Between the Severity of Neuropsychiatric Symptoms and Automatically Extracted Free‐Speech Features

**DOI:** 10.1002/alz70856_098535

**Published:** 2025-12-24

**Authors:** Zampeta‐Sofia Alexopoulou, Qingyue Li, Stefanie Köhler, Elisa Mallick, Johannes Tröger, Josef Priller, Annika Spottke, Björn Falkenburger, Melina Stark, Luca Kleineidam, Jens Wiltfang, Inga Zerr, Frank Jessen, Emrah Düzel, Michael Wagner, Christoph Laske, Stefan Teipel, Valeria Manera, Alexandra König

**Affiliations:** ^1^ CoBTeK (Cognition‐Behaviour‐Technology) Research Lab, Université Côte d'Αzur, Nice, France; ^2^ Rostock University Medical Center, Rostock, Germany; ^3^ German Center for Neurodegenerative Diseases (DZNE), Rostock, MV, Germany; ^4^ ki:elements GmbH, Saarbrücken, Germany; ^5^ Department of Psychiatry and Psychotherapy, School of Medicine and Health, Technical University of Munich, and German Center for Mental Health (DZPG), Munich, Germany; ^6^ Department of Psychiatry and Psychotherapy, Charité‐Universitaetsmedizin Berlin, Berlin, Germany; ^7^ University of Edinburgh and UK DRI, Edinburgh, United Kingdom; ^8^ German Center for Neurodegenerative Diseases (DZNE), Berlin, Germany; ^9^ Department of Neurology, University of Bonn, Bonn, Germany; ^10^ German Center for Neurodegenerative Diseases (DZNE), Venusberg‐Campus 1, 53127, Bonn, Germany; ^11^ University Hospital Carl Gustav Carus, Technische Universität Dresden, Dresden, Germany; ^12^ German Center for Neurodegenerative Diseases (DZNE), Dresden, Germany; ^13^ Department for Cognitive Disorders and Old Age Psychiatry, University Hospital Bonn, Bonn, Germany; ^14^ German Center for Neurodegenerative Diseases (DZNE), Bonn, Germany; ^15^ Department of Old Age Psychiatry and Cognitive Disorders, University Hospital Bonn and University of Bonn, Bonn, Germany; ^16^ German Center for Neurodegenerative Diseases (DZNE), Göttingen, Germany; ^17^ Department of Psychiatry and Psychotherapy, University Medical Center Goettingen, University of Goettingen, Goettingen, Germany; ^18^ Neurosciences and Signaling Group, Institute of Biomedicine (iBiMED), Department of Medical Sciences, University of Aveiro, Aveiro, Portugal; ^19^ University Medical Center, Georg August University, Goettingen, Germany; ^20^ German Center for Neurodegenerative Diseases (DZNE), Goettingen, Germany; ^21^ Department of Psychiatry and Psychotherapy, Medical Faculty, University of Cologne, Cologne, Germany; ^22^ Excellence Cluster on Cellular Stress Responses in Aging‐Associated Diseases (CECAD), Faculty of Medicine and University Hospital Cologne, Cologne, Germany; ^23^ Institute of Cognitive Neuroscience, University College London (UCL), London, United Kingdom; ^24^ German Center for Neurodegenerative Diseases (DZNE), Magdeburg, Germany; ^25^ Institute of Cognitive Neurology and Dementia Research (IKND), Otto‐von‐Guericke University, Magdeburg, Sachsen Anhalt, Germany; ^26^ German Center for Neurodegenerative Diseases (DZNE), Tübingen, Germany; ^27^ Section for Dementia Research, Hertie Institute for Clinical Brain Research and Department of Psychiatry and Psychotherapy, University of Tübingen, Tübingen, Germany; ^28^ Centre Hospitalier Universitaire, Clinique Gériatrique du Cerveau et du Mouvement, Centre Mémoire de Ressources et de Recherche, Nice, France

## Abstract

**Background:**

Neuropsychiatric symptoms (NPS) can precede cognitive decline in Alzheimer's Disease and serve as prognostic factors of disease progression. Assessing NPS in clinical practice is challenging due to time constraints, subjectivity and limited adaptability of tools to early‐stage cognitive decline. Advancements in automatic speech analysis may enable objective characterization of NPS, while structural brain morphometry associated with speech features offers insights into the underlying mechanisms of NPS. This study explored associations between extracted speech features, gold‐standard NPS assessments and volumetric brain measures.

**Method:**

Within the PROSPECT‐AD project, data were obtained from the German DELCODE and DESCRIBE cohorts. Analysis included *N* = 30 healthy controls and *N* = 44 participants with Subjective Cognitive Decline (SCD)/Mild Cognitive Impairment (MCI) with NPS, assessed by the Geriatric Depression Scale and the Neuropsychiatric Inventory. Participants answered a free‐speech question (“Can you describe a positive event in your life?”). Acoustic features (spectral/temporal/frequency/energy variables) and linguistic (i.e. lexical richness, syntactic complexity) were automatically extracted. Residuals from regression models (adjusted for age, sex, MMSE) were used to compute Spearman rank correlations between speech features and clinical scores measuring depression, apathy, anxiety and agitation. In the SCD/MCI group with NPS (*N* = 21), adjusted correlations were computed between speech features and baseline volumetric measures of regions of interest (ROI). Hypothesis‐driven mediation analysis between speech features, clinical scales and ROI volumes is ongoing.

**Results:**

We found significant associations (*p* < 0.05) between the severity of NPS and acoustic/linguistic markers, though these did not survive multiple comparisons corrections (Figure 1). For example, anxiety positively correlated with the sum and duration of pauses, while depression positively correlated with vocal tremor. In the SCD/MCI group with NPS, speech features correlated with volumes in key ROIs, especially related to emotion regulation (i.e. amygdala, insular cortex) (Figure 2).

**Conclusion:**

Our exploratory findings indicate that (mainly acoustic) markers derived from free speech are associated with the severity of NPS as measured by clinical scales and with regional brain volumes in participants with NPS. These dual associations highlight the potential of speech analysis as a non‐invasive, objective tool to assess NPS in early‐stage cognitive decline.